# Downregulated microRNA-129-5p by Long Non-coding RNA NEAT1 Upregulates PEG3 Expression to Aggravate Non-alcoholic Steatohepatitis

**DOI:** 10.3389/fgene.2020.563265

**Published:** 2021-01-26

**Authors:** Zhi Zhang, Huiqing Wen, Bangjian Peng, Jun Weng, Fanhong Zeng

**Affiliations:** ^1^Department of Hepatobiliary Surgery, The Fifth Affiliated Hospital, Southern Medical University, Guangzhou, China; ^2^Department of Hepatobiliary Surgery II, Zhujiang Hospital, Southern Medical University, Guangzhou, China

**Keywords:** long non-coding RNA NEAT1, microRNA-129-5p, paternally expressed gene 3, hepatic stellate cells, methionine–choline deficient, non-alcoholic steatohepatitis

## Abstract

Long non-coding RNAs (lncRNAs) have recently emerged as inflammation-associated biological molecules with a specific role in the progression of liver fibrosis conditions including non-alcoholic steatohepatitis (NASH). The aim of this study was to elucidate the effects of lncRNA nuclear enriched abundant transcript 1 (NEAT1), microRNA-129-5p (miR-129-5p), and paternally expressed gene 3 (PEG3) on the biological activities of hepatic stellate cells (HSCs) subjected to NASH. First, microarray-based analysis revealed upregulated PEG3 in NASH. Liver tissues from mice fed a methionine–choline-deficient (MCD) diet exhibited increased expression of NEAT1 and PEG3 along with lower miR-129-5p expression. A series of *in vitro* and *in vivo* assays were then performed on HSCs after transfection with shPEG3, miR-129-5p mimic, or treatment with pyrrolidine dithiocarbamate (PDTC), an inhibitor of the nuclear factor-kappa B (NF-κB) signaling pathway. Results confirmed the alleviated fibrosis by restoring miR-129-5p, while depleting PEG3 or NEAT1, as evidenced by the inactivation of HSCs. To sum up, NEAT1 can bind specifically to miR-129-5p and consequently regulate miR-129-5p and PEG3 expression in relation to the HSC activation occurring in NASH. Thus, NEAT1-targeted inhibition against miR-129-5p presents a promising therapeutic strategy for the treatment of NASH.

## Introduction

Non-alcoholic steatohepatitis (NASH) is a condition that is marked by hepatic inflammation and necrosis accompanied by fat storage and fatty deposition in liver parenchymal cells, which accounts for 25% of the causes of hepatocellular carcinoma (HCC) (Charrez et al., 2016). NASH can potentially progress to cirrhosis featured with decompensation, further resulting in liver transplantation or fatality in some cases (Neuschwander-Tetri, 2017). Hitherto, no effective pharmacological therapies or non-invasive diagnostic approaches have been identified for NASH, despite its increasing prevalence (Schuppan and Schattenberg, 2013). A functional study has pointed out that an advanced-stage liver fibrosis is a predictor of future morbidity from NASH, thus calling for early diagnosis and timely intervention (Stal, 2015). In light of this, exploring possible therapeutic strategies for NASH targeting inflammation and fibrosis is an emerging research topic (Musso et al., 2016).

Notably, long non-coding RNAs (lncRNAs) have been highlighted as potential regulators of liver fibrosis (Zhang et al., 2018). For instance, lncRNA Alu-mediated p21 transcriptional regulator has been identified as a biological regulator in hepatofibrogenesis (Yu et al., 2017). In particular, lnc18q22.2 has emerged as a regulator of hepatocyte viability in the liver of NASH patients (Atanasovska et al., 2017). Also, lncRNA nuclear enriched abundant transcript 1 (NEAT1), a main component of paraspeckles, has been demonstrated to alleviate fibrosis in the context of diabetic nephropathy (Huang et al., 2019). Furthermore, the expression of NEAT1 can be detected in multiple tumor types and other non-tumor diseases (Yu et al., 2017; Prinz et al., 2019). More importantly, NEAT1 plays an important role in a rat model of non-alcoholic fatty liver disease (NAFLD) involving the mammalian target of rapamycin/S6 kinase beta-1 signaling pathway (Wang, 2018). It was also reported that NEAT1 could affect HCC through interaction with microRNA-129-5p (miR-129-5p) (Fang et al., 2017), which has also been proposed as a biomarker of liver fibrosis responsible for the activation of hepatic stellate cells (HSCs) and collagen synthesis (Chen et al., 2018). NEAT1 also epigenetically silences miR-129-5p expression by promoting the DNA methylation of the CpG island in the miR-129 gene in breast cancer (Lo et al., 2016). Additionally, the activation and survival of HSCs are subject to regulation by the nuclear factor-kappa B (NF-κB) signaling pathway, which is activated in response to liver injury and inflammation, thus forming a mechanistic link between chronic liver injury, fibrosis, and HCC together (He and Karin, 2011; Luedde and Schwabe, 2011). Furthermore, miR-129-5p is highly expressed in brain damage and hepatic cancer in mice (Xiong et al., 2020). Paternally expressed gene 3 (PEG3) is a paternally expressed imprinted gene dependent on DNA methylation to establish and maintain its allelic expression and can mediate gene transcription in association with cellular metabolism (McNamara et al., 2018). PEG3 is also extensively expressed in various tissues of both human and mice.^1^ Myocardial stem cells with PEG3 positive expression are inclined to differentiate into fibroblasts (Yaniz-Galende et al., 2017). Bioinformatics prediction suggested PEG3 to be a target gene of miR-129-5p, and indeed, ectopic expression of PEG3 was found in mice suffering from NASH. In the current study, we probed in detail the functioning of a NEAT1/miR-129-5p/PEG3 axis in NASH and identified the underlying mechanisms with the NF-κB signaling pathway, which may guide research toward much-needed new approaches for the treatment for NASH.

## Materials and Methods

### Ethics Statement

The study was approved by the Institutional Animal Care and Use Committee of the Fifth Affiliated Hospital, Southern Medical University.

### Bioinformatics Prediction

The Gene Expression Omnibus (GEO) database^2^ was searched to download NASH-related microarray data (GSE35961) and an annotated probe document following the Affymetrix Mouse Genome 430 2.0 Array test (Mouse430_2). The microarray data consisted of 12 samples [untreated liver samples collected from healthy mice aged 8 weeks; liver samples from mice fed with methionine–choline-deficient (MCD) and high fat (HF) diet; liver samples from mice treated with MCD, HF, and 0.1% metformin]. Normal samples were used as controls, and NASH samples were used as treatment groups for differential analysis. Background correction and normalization processing were performed by means of the Affy installation package of R software (Fujita et al., 2006). An Empirical Bayes method of the linear model in the Limma installation package, combined with traditional *t*-test, served to filter non-specific expression data and screen differentially expressed genes (Smyth, 2004). The regulatory miRNA for the target gene in mouse was predicted on the microRNA.org database,^3^ miRWalk database,^4^ and TargetScan.^5^ Venn diagram of prediction results from the three databases was established on http://bioinformatics.psb.ugent.be/webtools/Venn/, and the intersection of the three databases was used for subsequent experiments.

### Establishment of Mouse Model With NASH

A total of 42 specific-pathogen-free (SPF) level C57BL/6J male mice (age: 6 weeks, weight: 20–30 g) were purchased from SPF (Beijing) Biotechnology Co., Ltd. (Beijing, China). All mice were housed in SPF-level animal laboratory with humidity of 60–65% at 22–25°C for 7 days of adaptive feeding. Free access to drinking water was guaranteed in an environment with 12:12 h light/dark cycle. Six randomly selected mice were fed with normal diet (Harlan Teklad, TD94149) and the remaining 36 mice were fed with MCD diet (Harlan Teklad, TD90262) to establish the NASH model. The success of modeling was evaluated according to the following criteria: (1) general condition of mice was observed, including food intake, water drinking, behavior, movement, mental state, fur, weight, urine, and stool; (2) liver pathology changes were detected, including steatosis, inflammation, and fibrosis; (3) serum levels of alanine aminotransferase (ALT) and aspartate aminotransferase (AST) were determined.

Adenoviruses expressing the following plasmids were packaged: short hairpin RNA (shRNA) against NEAT1 (Ad-shNEAT1), shRNA against NEAT1mu (Ad-shNEAT1mu) using mutant form of NEAT1, shRNA against PEG3 (Ad-shPEG3), shRNA against PEG3mu (Ad-shPEG3mu) using mutant form of PEG3, NEAT1 (Ad-NEAT1), or PEG3 (Ad-PEG3). Adenovirus (1 × 10^9^ PFU/100 μl) or miR-129-5p antagomir (20 nM) was introduced into C57BL/6 mice (at least aged 8 weeks) via tail injection (twice a week, 6 weeks in total). The 30 mice for modeling were randomly assigned to five groups (*n* = 6 each): mice fed with MCD diet only, mice fed with MCD diet and treated delivered with ad-shNEAT1, mice fed with MCD diet and delivered with ad-shPEG3, mice fed with MCD diet and delivered with ad-control, and mice fed with MCD diet and delivered with both ad-shNEAT1 and ad-shPEG3. We randomly classified all mice into different groups based on weekly assessment post-modeling and then randomly selected mice from each treatment group for subsequent experiments. The selected mice were euthanized with 1% sodium pentobarbital, followed by liver resection for subsequent analysis.

### Sample Collection

Mice were fasted the day before sample collection. After fasting, whole blood was extracted from the abdominal aorta and mice were euthanized by cervical dislocation. The blood was allowed to stand at room temperature for 0.5 h. The supernatant was obtained through centrifugation at 800 × *g* for 15 min, followed by another round of centrifugation at 12,000 × *g* for 15 min. Serum levels of ALT and AST were measured with an automatic biochemical analyzer. The liver was immediately resected from the mice. Liver tissues in small pieces were sliced from the edge of the greatest liver lobe, fixed by immersion in 4% formaldehyde, dehydrated conventionally, embedded with paraffin, and sliced. Hematoxylin–eosin (HE) staining, Oil Red O staining, and Masson staining were then conducted. The sections were analyzed using ImageJ, and the proportion of the pathologically changed area in sections was calculated.

### HE Staining

The liver slices (4 μm in thickness) were baked at 60°C for 1 h and dewaxed with xylene, dehydrated by gradient alcohol, and washed in buffer. The slices were then stained with hematoxylin for 10 min, whereupon 1% hydrochloric acid in ethanol was used for differentiation for 20 s. The slices were then placed in 1% ammonia for 30 s to return the color to blue, followed by eosin staining for 3 min, whereupon the sections were dehydrated with gradient ethanol and cleared in xylene. Finally, the mounted and sealed slices were observed under an ordinary optical microscope (Olympus, Tokyo, Japan). The histological scoring of liver damage referred to the NASH activity score (Li et al., 2018).

### Oil Red O Staining

Liver tissues were cut into 6-μm-thick cryostat sections. After being dried by air cooler, sections were fixed in 10% formaldehyde solution-1% CaCl_2_ for 15 min, rinsed with 60% isopropyl alcohol for 2 min, and stained with Oil Red O working solution for 10 min, followed by differentiation using 60% isopropyl alcohol. The sections were mounted with glycogelatin and then observed under an ordinary optical microscope (Olympus, Tokyo, Japan). The average optical density (OD) was analyzed using Image Pro-Plus image analysis software.

### Masson Staining

Five slices were obtained, treated with Ponceau for 2 min, and then immersed in 0.2% glacial acetic acid for 2 min, 5% molybdophosphoric acid aqueous solution for 2 min, 0.2% glacial acetic acid for 2 min, and finally methyl green solution for 3 min. Then, the slices were rinsed with 95% ethanol, followed by ethanol dehydration and xylene clearing. Under the microscope, blue-stained areas were enriched with collagen fiber and red-stained areas were healthy cellular matrix. Ten fields of view were randomly selected from each slice and analyzed by ImageJ software. The degree of liver fibrosis = fibrosis area/total area × 100%.

### HSC Sorting

Mouse primary HSCs were isolated following the steps as previously reported (Weiskirchen et al., 2017). The isolated HSCs were cultured with Dulbecco’s Modified Eagle Medium (DMEM) (Sigma-Aldrich Chemical Biotechnology Company, St Louis, MO, United States) containing 10% fetal bovine serum (FBS), 100 U/ml penicillin, and 100 μg/ml streptomycin. Immunocytochemistry (IHC) staining with antibody against α-smooth muscle actin (α-SMA) was performed to verify the successful isolation of HSCs.

### Cell Grouping and Transfection

Mouse HSCs in the logarithmic growth phase were cultured in DMEM containing 10% FBS. When cell confluence reached 40–60%, HSCs (5 × 10^3^ cells/well) were transfected with the following plasmids according to the instructions of the Lipofectamine 2000 kit (11668-027, Invitrogen Inc., Carlsbad, CA, United States): pcDNA3-shRNA-NC, pcDNA3, pcDNA3-shPEG3, miR-NC, miR-129-5p mimic, pcDNA3-NEAT1, pcDNA3-PEG3, or their respective NCs. Besides, cells were treated with dimethyl sulfoxide (DMSO) or pyrrolidine dithiocarbamate (PDTC, an inhibitor of the NF-κB signaling pathway) (2 mmol/l for 24 h). All plasmids and miR-129-5p mimic were purchased from Shanghai GenePharma Co., Ltd. (Shanghai, China).

### 5-Ethynyl-2′-Deoxyuridine Assay

At 48 h post-transfection, HSCs were seeded in a 24-well plate at a density of 5 × 10^5^ cells/ml (500 μl/well). HSCs were incubated with 5-ethynyl-2′-deoxyuridine (EdU) solution for 12 h and their proliferation was detected following instructions of Cell-Light^TM^ EdU *in vitro* Imaging Detection Kit (C10310-1, Guangzhou RiboBio Co., Ltd., Guangzhou, Guangdong, China).

### Luciferase Reporter Assay

The artificially synthesized PEG3 3′-untranslated region (3′UTR), as well as a form with mutation of potential miR-129-5p binding sites, were mutated and introduced into the pGL3-reporter (Promega Corp., Madison, WI, United States). The wild-type (pGL3-PEG3-WT) or mutant type (Mut) (pGL3-PEG3-Mut) was co-transfected into primary HSCs (5 × 10^3^ cells/well) with miR-129-5p mimic or miR-NC, respectively. Cells were harvested and lysed at 48 h after transfection, and luciferase activity was measured using Dual-Luciferase^TM^ Reporter Assay System (Promega Corp., Madison, WI, United States) on a Luminometer TD-20/20 detector (E5311, Promega Corp., Madison, WI, United States). The target relationship between NEAT1 and miR-129-5p was verified similarly. In brief, NEAT1 WT or Mut was inserted into the pGL3-reporter to construct pGL3-NEAT1-WT or pGL3-NEAT1-Mut, which were then co-transfected with miR-129-5p mimic or miR-NC, respectively, into primary HSCs, and the luciferase activities were then determined.

### Fluorescence *in situ* Hybridization

The subcellular localization of NEAT1 was analyzed by a fluorescence *in situ* hybridization (FISH) kit (Roche Applied Science, Germany). Probes of NEAT1 and lncRNA 18S were synthesized by RiboBio (Guangzhou, Guangdong, China). HSCs were fixed with 4% paraformaldehyde at room temperature for 10 min, followed by incubation overnight with digoxin-labeled NEAT1 probes. LncRNA 18S probe served as a cytoplasm control. The nucleus was then stained with 4′,6-diamidino-2-phenylindole (DAPI), and the cells were observed under a fluorescence microscope (Olympus, Tokyo, Japan).

### RNA Pull-Down Assay

RNA pull-down assay was carried out following procedures as reported previously (Wang et al., 2014). In brief, primary HSCs (5 × 10^3^ cells/well) were cultured for 2 days and then transfected with biotinylated miR-129-5p-wt (bio-miR-129-5p-wt, 40 nM) or biotinylated miR-129-5p-mut (bio-miR-129-5p-mut). At 48 h after transfection, cells were collected and lysed at room temperature for 10 min. The lysate was incubated with streptavidin magnetic beads (Thermo Fisher Scientific Inc., San Jose, CA, United States) at 4°C for 4 h, washed twice with lysis buffer, three times in low-salt buffer solution and once in high-salt buffer solution. RNA was extracted by TRIzol and the expression of NEAT1 was detected by transcription quantitative polymerase chain reaction (RT-qPCR).

### NF-κB Luciferase Assay

The day before transfection, HSCs were inoculated into a 96-well plate at a density of 5 × 10^3^ cells/well. Following the instructions of the Lipo2000 kit, NF-κB luciferase reporter (20 ng) (Promega Corp., Madison, WI, United States) and pRL-TK (5 ng) (Promega Corp., Madison, WI, United States) were co-transfected with shPEG3 or miR-129-5p mimic into HSCs, and the luciferase activity was determined.

### RNA Immunoprecipitation

The binding between NEAT1 and Argonaute2 (Ago2) and that between miR-129-5p and Ago2 was detected using an RNA immunoprecipitation (RIP) kit (Millipore, Temecula, CA, United States). Cells were collected and washed with pre-cooled phosphate buffer saline (PBS) and then lysed by rapid immunofilter paper assay (RIPA) lysis (Sigma-Aldrich Chemical Biotechnology Company, St Louis, MO, United States) in an ice bath for 5 min. The supernatant was obtained after centrifugation (14,000 rpm, 4°C, 10 min) and probed with rabbit anti Ago2 (ab32381, 1:50, Abcam Inc., Cambridge, MA, United States) for coprecipitation. RNA was extracted after protease K treatment, followed by RT-qPCR.

### RT-qPCR

Total RNA was extracted from collected HSCs according to the instructions provided by the TRIzol kit (Invitrogen Inc., Carlsbad, CA, United States). The first-strand cDNA was reversely transcribed using first-strand cDNA synthesis, following instructions of First Strand cDNA Synthesis Kit (Takara Co., Ltd., Dalian, Liaoning, China). The expression of the target gene was determined by real-time qPCR using the SYBR Premix Ex Taq kit (Takara Co., Ltd., Dalian, Liaoning, China) on an ABI Prism 7500 Fast Real-Time PCR System (Thermo Fisher Scientific Inc., Waltham, MA, United States) with U6 and β-actin as internal references. The relative expression of the target gene was calculated by the 2^–Δ^ ^Δ^ ^*Ct*^ method. The primer sequences are shown in Table 1.

**TABLE 1:** Primer sequences for reverse transcription quantitative polymerase chain reaction.

**Gene**	**Primer sequences**
miR-129-5p	F: 5′′-ACACTCCTTTTTGCGTCTGGGCTTGC-3′
	R: 5′-TGGTGTCGTGGAGTCG-3′
NEAT1	F: 5′-GTGGCTGTTGGAGTCGGTAT-3′
	R: 5′-TAACAAACCACGGTCCATGA-3′
PEG3	F: 5′-CTTCGCGGTCATTTCTGAGT-3′
	R: 5′-TTGTCCTTGCCGTACATCTTC-3′
Col1A1	F: 5′-GACGCCATCAAGGTCTACTG-3′
	R: 5′-ACGGGAATCCATCGGTCA-3′
α-SMA	F: 5′-TACTGCCGAGCCTGAGAT-3′
	R: 5′-GCTTCGTCGTATTCCTGTTT-3′
β-actin	F: 5′-CAGAAGGACTCGTACGTGGG-3′
	R: 5′-TTGGCCTTAGGGTTCAGGG-3′
U6	F: 5′-CTCGCTTCGGCAGCACA-3′
	R: 5′-AACGCTTCACGAATTTGCGT-3′

*miR, microRNA; NEAT1, nuclear enriched abundant transcript 1; PEG3, paternally expressed gene 3; Col1A1, collagen type I alpha 1; SMA, smooth muscle actin; F, forward; R, reverse.*

### Western Blot Analysis

Total protein was extracted from collected HSCs using RIPA lysis (Sigma-Aldrich Chemical Biotechnology Company, St Louis, MO, United States). Nucleoprotein was extracted using Qproteome Cell Compartment Kit (QIAGEN, Dusseldorf, Germany). The proteins (20 μg) were separated using 10% sodium dodecyl sulfate-polyacrylamide gel electrophoresis (SDS-PAGE) and transferred onto a polyvinylidene difluoride (PVDF) membrane (Millipore, Temecula, CA, United States). The membrane was blocked in 5% skimmed milk for 1 h at room temperature, washed once with PBS, and incubated with the following primary antibodies, all provided by Abcam Inc. (Cambridge, MA, United States) at 4°C overnight: rabbit anti PEG3 (ab99252, 1:1,000), α-SMA (ab223068, 1:500), collagen type I alpha 1 (Col1A1) (ab34710, 1:1,000), glyceraldehyde-3-phosphate dehydrogenase (GAPDH) (ab9485, 1:2,500), p50 (ab131546, 1:500), p65 (ab16502, 1:1,000), and H3 (ab1791, 1:1,000). After three PBS washes at room temperature, the membrane was incubated with horseradish peroxidase (HRP)-labeled immunoglobulin G (IgG) antibody (ab97051, 1:200, Abcam Inc., Cambridge, MA, United States) at 37°C for 1 h, followed by three PBS washes (5 min each time) at room temperature. The membrane was then immersed in enhanced chemiluminescence (ECL) solution (Pierce Chemical, Dallas, YA, United States) at room temperature for 1 min for development. ImageJ software was used to analyze the gray value intensity of the bands of target genes.

### Immunofluorescence Assay

Hepatic stellate cells were cultured on a coverslip (18 mm) for 24 h and fixed in solution (acetic acid/ethanol = 1:3) at −20°C for 5 min. HSCs were then blocked with 5% goat serum at room temperature for 1 h and incubated with primary antibodies provided by Abcam Inc., (Cambridge, MA, United States) at 4°C overnight: rabbit anti α-SMA (ab223068, 1:50) or collagen I (ab34710, 1:500). Following two PBS washes, HSCs were incubated with fluorescein-labeled secondary antibody (1:50, Dianova Hamburg, Germany) for 1 h at room temperature in the dark, and nuclei were then stained with DAPI at room temperature in the dark for 30 min. The cells were mounted and observed under confocal laser scanning microscopy (Olympus, Tokyo, Japan) at 488 and 364 nm.

### Statistical Analysis

SPSS 21.0 software (IBM Corp., Armonk, NY, United States) was applied for data analysis. The measurement data was expressed as mean ± standard deviation. Comparison between two groups was conducted by independent sample *t*-test with the Welch correction. Shapiro–Wilk test was employed for testing normal distribution, and one-way analysis of variance was used for comparison of normally distributed data among multiple groups. Least significant difference analysis was used for pairwise comparison among multiple groups. Data that failed to follow normal distribution were compared using non-parametric Kruskal–Wallis test. Values of *p* < 0.05 were considered statistically significant.

## Results

### miR-129-5p/PEG3 Axis May Be Involved in Liver Fibrosis of NASH

**FIGURE 1:**
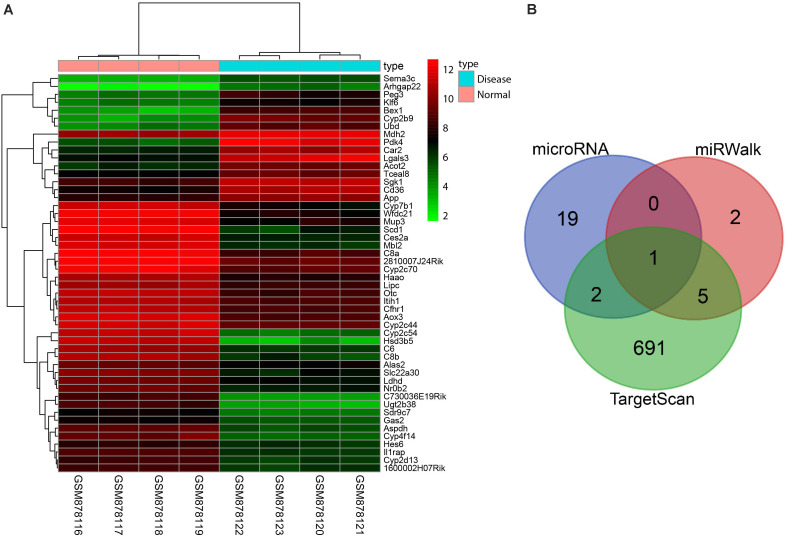
Bioinformatics analysis shows that PEG3 is highly expressed and can be targeted by miR-129-5p. **(A)** Heap map displaying the top 50 differentially expressed genes in GSE35961. The X-axis represents sample number. The Y-axis represents the gene name. Each small square in the graph represents the expression of a gene in a sample; the left dendrogram represents the gene clustering, while the upper dendrogram represents the sample clustering. The right histogram shows the color gradation. **(B)** Venn diagram illustrating miRNAs that can target PEG3. PEG3, paternally expressed gene 3; GSE, Gene Expression Omnibus Series.

Initially, we used the R software for analysis of expression data from GSE35961 to obtain differentially expressed genes in untreated mice and NASH model mice. As shown in Figure 1A, PEG3 was highly expressed in the NASH mice. Then, we searched for miRNAs that might target PEG3 using databases available from microRNA.org, miRWalk, and TargetScan. Venn analysis showed only one miRNA, miR-129-5p, to be present in all three databases (Figure 1B). PEG3 can activate the NF-κB signaling pathway by interacting with tumor necrosis factor receptor-associated factor 2 (TRAF2) (Relaix et al., 1998). Meanwhile, the NF-κB signaling pathway is closely correlated with liver fibrosis in NASH (Xiao et al., 2014). Additionally, miR-129-5p has been proven to negatively regulate the fibrosis process (Xiao et al., 2015). Thus, we proposed the reasonable hypothesis that the miR-129-5p/PEG3 axis might play a part in liver fibrosis in NASH through regulating the NF-κB signaling pathway.

### The Mouse Model of NASH Was Successfully Established

**FIGURE 2:**
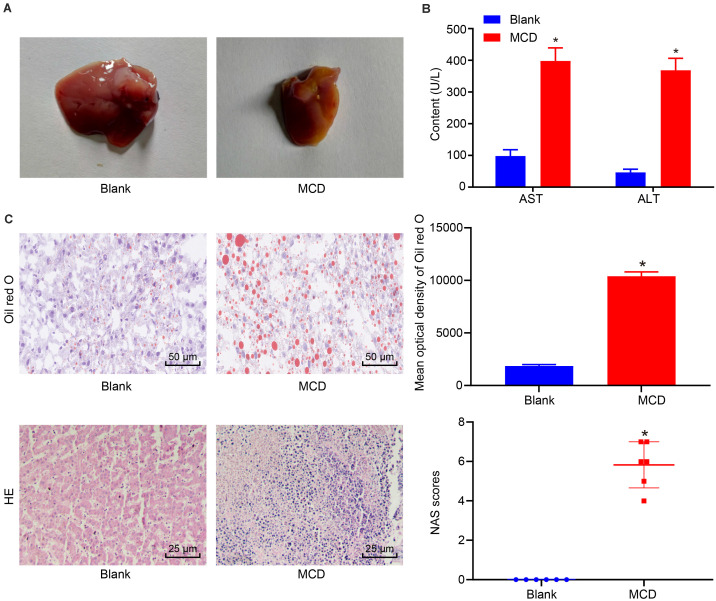
Successful establishment of the mouse NASH model. **(A)** Representative images of liver resected from mice with normal diet and 5 weeks of MCD diet. **(B)** Serum levels of ALT and AST in mice on normal and 5-week MCD diets. **(C)** Pathological changes of liver tissue in mice on normal and 5-week MCD diets detected by Oil Red O staining (×200) (the upper diagram) and HE staining (×400) (the lower diagram). *p < 0.05 vs. the blank group (mice on normal diet). All data were measurement data and expressed as mean ± standard deviation. Comparison between two groups was analyzed using unpaired *t*-test. *N* = 6. NASH, non-alcoholic steatohepatitis; MCD, methionine–choline deficient; ALT, alanine aminotransferase; AST, aspartate aminotransferase; HE, hematoxylin–eosin.

Subsequently, we established a murine model of NASH by feeding mice with MCD diet. No mice died during the modeling phase. Compared with mice on normal diet, mice on the MCD diet had matted hair, lower body weight, and showed less movement and apathetic behavior. Besides, mice on normal diet had glossy and soft livers of dark red color with sharp edges and normal density without swelling. As for mice on MCD diet, livers shown increased swelling, which had blunt edges and were dark yellow in color. We observed yellow fatty plaques, and the liver samples floated on the surface of the neutral formalin solution, suggesting decreased density (Figure 2A). Moreover, MCD diet caused significantly higher levels of ALT and AST in serum when compared with normal diet (Figure 2B, *p* < 0.05). HE staining showed normal morphological structure of liver tissue from mice on normal diet. However, mice on 5-week MCD diet had fat vacuoles in the hepatic lobule, ballooning degeneration in hepatic cells, and focal infiltration of inflammatory cells, all indicating the occurrence of liver fibrosis (Figure 2C). According to Oil Red O staining, fat particles were detected in hepatic cells of MCD diet mice, but not in control mice (Figure 2C). The aforementioned findings demonstrate successful establishment of the mouse model of NASH.

### PEG3 Is Highly Expressed in HSCs With Fibrosis

**FIGURE 3:**
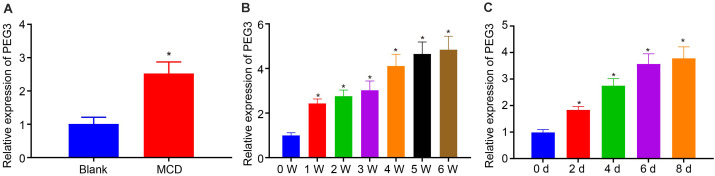
PEG3 expression is upregulated in liver fibrosis of NASH. **(A)** Relative expression of PEG3 in liver tissues of mice on normal and 5-week MCD diets. **(B)** Relative expression of PEG3 in HSCs at different time points of mice on MCD diet from weeks 0 to 6. **(C)** Relative expression of PEG3 in HSCs at different time points of mice on normal diet from days 0 to 8. *p < 0.05 vs. the blank group (mice on normal diet) or the MCD group (mice on MCD diet) at 0 week or the blank group (mice on normal diet) on day 0. All data were measurement data and expressed as mean ± standard deviation. Comparison between two groups was analyzed using independent sample *t*-test. One-way analysis of variance was used for data comparison among multiple groups. The experiment was independently repeated three times. *N* = 6. HSCs, hepatic stellate cells.

To explore whether PEG3 participated in liver fibrosis of NASH, we first determined the expression of PEG3 in NASH mice induced by MCD diet. Significantly higher PEG3 expression was detected in the liver of NASH mice (Figure 3A, *p* < 0.05). Furthermore, the expression of PEG3 in primary HSCs isolated from NASH mice increased over the duration of the MCD induction (Figure 3B, *p* < 0.05). Meanwhile, the level of PEG3 in primary HSCs from normal mice was also gradually increased from day 1 to day 8 (Figure 3C, *p* < 0.05). These findings suggest that PEG3 is upregulated in activated HSCs in association with liver fibrosis of NASH.

### Silencing PEG3 Inhibits the Activation of HSCs *in vitro*

**FIGURE 4:**
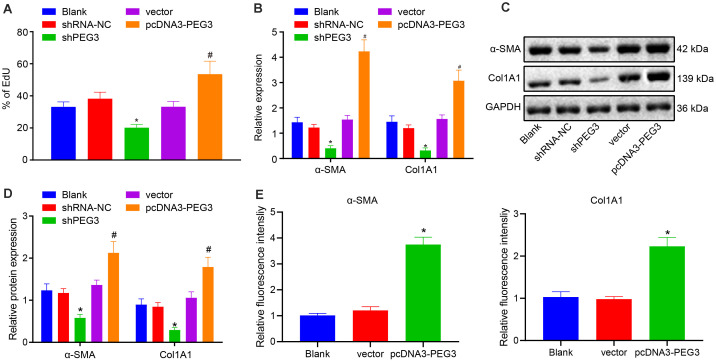
Depletion of PEG3 inactivates HSCs *in vitro*. **(A)** HSC proliferation after transfection detected by EdU assay. **(B)** Relative mRNA levels of α-SMA and Col1A1 in HSCs after transfection determined by RT-qPCR. **(C)** Protein bands of α-SMA, Col1A1, and GAPDH in HSCs after transfection. **(D)** Relative protein levels of α-SMA and Col1A1 normalized to GAPDH in HSCs after transfection determined by western blot analysis. **(E)** Quantitative analysis on α-SMA and Col1A1 immunofluorescence intensity in primary HSCs. *p < 0.05 vs. the blank group (HSCs without transfection). ^#^*p* < 0.05 vs. the vector group (HSCs treated with vector). All data were measurement data and expressed as mean ± standard deviation. One-way analysis of variance was used for data comparison among multiple groups. The experiment was independently repeated three times. *N* = 6. α-SMA, α-smooth muscle actin; Col1A1, collagen type I alpha 1; RT-qPCR, reverse transcription quantitative polymerase chain reaction; GAPDH, glyceraldehyde-3-phosphate dehydrogenase.

Primary HSCs were isolated from normal mice and cultured for 4 days and were then transfected with shPEG3 or pcDNA-PEG3. EdU assay showed that cell proliferation was inhibited when PEG3 was silenced in HSCs (Figure 4A, *p* < 0.05). Moreover, silencing PEG3 led to reduced expression of α-SMA and Col1A1 (Figures 4B–D, *p* < 0.05). Opposite results were observed when PEG3 was overexpressed in HSCs (Figures 4A–D, *p* < 0.05). Immunofluorescence staining also revealed that overexpression of PEG3 could increase the expression of α-SMA and Col1A1 (Figure 4E, *p* < 0.05). Therefore, downregulating PEG3 exerts inhibitory effects on the activation of HSCs *in vitro*.

### The Activation Effects of PEG3 on HSCs Are Negatively Regulated by miR-129-5p

**FIGURE 5:**
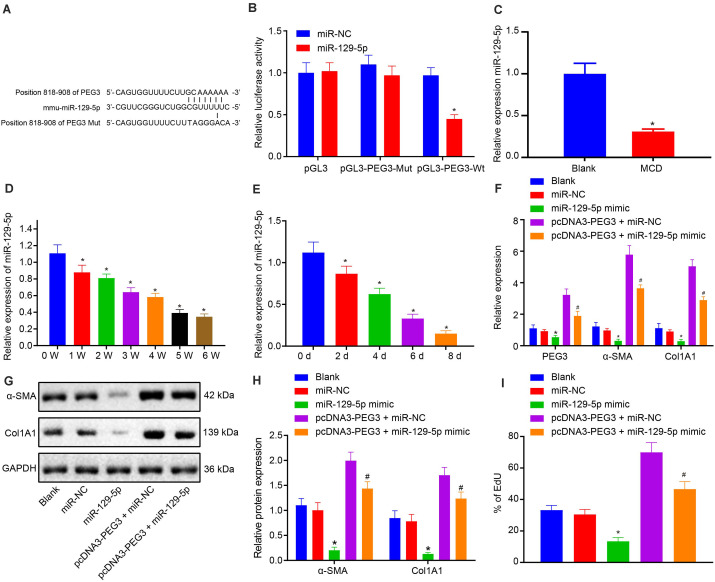
miR-129-5p negatively regulates the expression of PEG3 in HSCs. **(A)** Binding sequences between PEG3 and miR-129-5p. **(B)** Relative luciferase activity of pGL3-PEG3-WT and pGL3-PEG3-Mut. **(C)** Relative expression of miR-129-5p in liver tissues from mice on 5-week MCD diet (*n* = 6). **(D)** Relative expression of miR-129-5p in HSCs of mice on MCD diet from weeks 0 to 6. **(E)** Relative expression of miR-129-5p in HSCs of mice on normal diet from days 0 to 8. **(F)** Relative mRNA expression of PEG3, α-SMA, and Col1A1 after transfection determined by RT-qPCR. **(G)** Protein bands of α-SMA, Col1A1, and GAPDH after transfection. **(H)** Relative protein expression of α-SMA and Col1A1 normalized to GAPDH after transfection determined by western blot analysis. **(I)** HSC proliferation of mice on normal diet detected by EdU assay. *p < 0.05 vs. the blank group (mice on normal diet). ^#^*p* < 0.05 vs. the pcDNA3-PEG3 + miR-NC group (HSCs transfected with pcDNA3-PEG3 + miR-NC). All data were measurement data and expressed as mean ± standard deviation. Comparison between two groups was analyzed using unpaired *t*-test. One-way analysis of variance was used for data comparison among multiple groups. The experiment was independently repeated three times. *N* = 6. Wt, wild-type; Mut, mutant; EdU, 5-ethynyl-2′-deoxyuridine; NC, negative control.

Bioinformatics analysis predicted that miR-129-5p could bind to 3′UTR of PEG3 (Figure 5A). The luciferase reporter assay revealed that miR-129-5p could significantly reduce the luciferase activity of PEG3 3′UTR WT (*p* < 0.05), while there was no obvious effect on the luciferase activity of PEG3 3′UTR Mut (*p* > 0.05, Figure 5B), confirming that miR-129-5p could target and downregulate PEG3. Then, we proceeded to investigate the role of miR-129-5p in the regulatory mechanism of PEG3 in the liver. RT-qPCR showed that miR-129-5p expression was diminished in the liver of NASH mice (*p* < 0.05, Figure 5C) and exhibited a downward changing tendency over time in HSCs both from NASH mice and normal mice (*p* < 0.05, Figures 5D,E). Besides, overexpression of miR-129-5p significantly reduced the expression of PEG3 in HSCs (*p* < 0.05, Figure 5F). Moreover, miR-129-5p could inhibit HSC proliferation (*p* < 0.05, Figure 5I) and attenuated the high levels of α-SMA and collagen I that were induced by PEG3 (*p* < 0.05, Figures 5F–H). Therefore, we conclude that miR-129-5p negatively regulates the activation of HSCs caused by PEG3.

### NEAT1 Regulates PEG3 Expression by Binding to miR-129-5p

**FIGURE 6:**
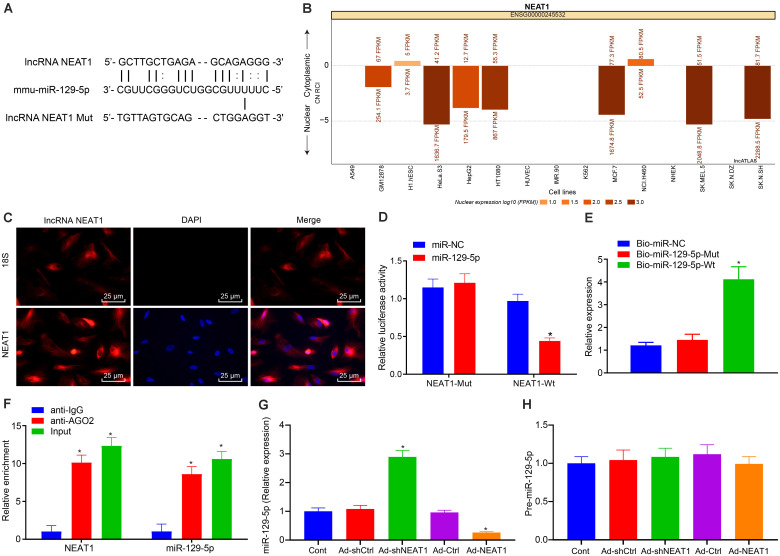
NEAT1 binds to miR-129-5p for regulation of PEG3 expression. **(A)** Binding sequences between NEAT1 and miR-129-5p. **(B)** Subcellular localization of NEAT1 analyzed on http://lncatlas.crg.eu/. **(C)** Subcellular localization of NEAT1 detected by FISH (×400). **(D)** Relative luciferase activity of NEAT1 WT 3′UTR or NEAT1 MUT 3′UTR. **(E)** Relative expression of NEAT1 in the presence of Bio-miR-129-5p-Mut or Bio-miR-129-5p-Wt detected by RNA pull-down. **(F)** Relative enrichment of Ago2 caused by NEAT1 and miR-129-5p detected by RIP assay. **(G)** The expression of miR-129-5p in each group detected by qRT-PCR. **(H)** The expression of precursor miR-129-5p in each group detected by qRT-PCR. *p < 0.05 vs. the miR-NC or Bio-miR-NC or anti-IgG group (cells in the presence of miR-NC or Bio-miR-NC or anti-IgG). All data were measurement data and expressed as mean ± standard deviation. One-way analysis of variance was used for data comparison among multiple groups. The experiment was independently repeated three times. *N* = 6. NEAT1, nuclear enriched abundant transcript 1; FISH, fluorescence *in situ* hybridization; Mut, mutant; Wt, wild-type; Ago2, Argonaute2; RIP, RNA immunoprecipitation; IgG, immunoglobulin G.

There is considerable evidence that lncRNAs can regulate the expression of miRNAs. We performed a bioinformatics analysis to identify upstream mechanisms that can regulate PEG3 during the process of liver fibrosis and discovered a targeting relationship between NEAT1 and miR-129-5p (Figure 6A). The website http://lncatlas.crg.eu/indicated that NEAT1 expression could be detected both in the nucleus and cytoplasm (Figure 6B), which was verified by FISH (Figure 6C). The luciferase reporter assay demonstrated that miR-129-5p transfection could weaken the luminescent signal of NEAT1 WT 3′UTR (*p* < 0.05) but exerted no significant effects on the luciferase activity of NEAT1 Mut 3′UTR (*p* > 0.05, Figure 6D). RNA pull-down further indicated that Bio-miR-129-5p-Wt could decrease the expression of NEAT1 (*p* < 0.05), while no significant changes of NEAT1 expression were found under induction of Bio-miR-129-5p-Mut (*p* > 0.05) in comparison to Bio-NC (Figure 6E), suggesting a direct interaction between miR-129-5p and NEAT1. Since miRNAs can inhibit the expression of their target genes by forming RNA-induced silencing complex (RISC) with Ago2, we performed the RIP assay, which showed that Ago2 antibody significantly enriched NEAT1 and miR-129-5p (*p* < 0.05, Figure 6F). These results together indicated that miR-129-5p could be targeted, inhibited, and degraded by NEAT1, in which NEAT1 might interact with miR-129-5p. Therefore, results implied that NEAT1 can target miR-129-5p by binding to Ago2. We further investigated if the expression of precursor miR-129-5p was regulated by NEAT1, finding that NEAT1 negatively regulated the expression of miR-129-5p (Figure 6G) but had little effect on the expression of precursor miR-129-5p (Figure 6H). From the aforementioned findings, we conclude that NEAT1 can affect the expression of miR-129-5p to regulate the expression of PEG3. At the same time, we found that miR-129-5p could inhibit the expression of NEAT1 and also inhibit the expression of downstream gene PEG3 (Supplementary Figure 1), further indicating that NEAT1 regulates the expression of PEG3 by binding to miR-129-5p.

### NEAT1 Activates HSCs via the miR-129-5p/PEG3 Axis

**FIGURE 7:**
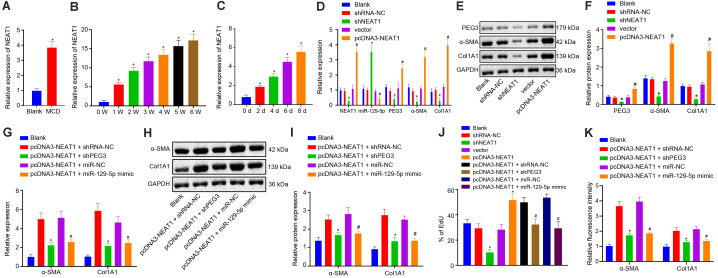
NEAT1 leads to activation of HSCs via the miR-129-5p/PEG3 axis. **(A)** Relative expression of NEAT1 in liver tissue of mice at 5 weeks post-modeling. **(B)** Relative expression of NEAT1 in HSCs of mice on MCD diet from weeks 0 to 6; ^∗^*p* < 0.05 vs. mice at 0 week. **(C)** Relative expression of NEAT1 in primary HSCs of mice on normal diet from days 0 to 8; ^∗^*p* < 0.05 vs. mice on day 0. **(D)** Relative expression of NEAT1, miR-129-5p, PEG3, α-SMA, and Col1A1 determined by RT-qPCR. **(E)** Protein bands of PEG3, α-SMA, Col1A1, and GAPDH. **(F)** Relative protein levels of PEG3, α-SMA, and Col1A1 normalized to GAPDH determined by western blot analysis. In panels **(D–F)**, *p < 0.05 vs. the blank, shRNA-NC, or vector group (HSCs without any treatment or in the presence of shRNA-NC or vector). **(G)** Relative expression of α-SMA and Col1A1 in the presence of pcDNA3-NEAT1 determined by RT-qPCR. **(H)** Protein bands of α-SMA, Col1A1, and GAPDH in the presence of pcDNA3-NEAT1. **(I)** Relative expression of α-SMA and Col1A1 in the presence of pcDNA3-NEAT1 normalized to GAPDH determined by western blot analysis. In panels **(G–I)**, *p < 0.05 vs. the pcDNA3-NEAT1 + shRNA-NC group (HSCs in the presence of pcDNA3-NEAT1 + shRNA-NC); ^#^*p* < 0.05 vs. the pcDNA3-NEAT1 + miR-NC group (HSCs in the presence of pcDNA3-NEAT1 + miR-NC). **(J)** HSC proliferation of mice on normal diet detected by EdU assay; *p < 0.05 vs. the shRNA-NC or vector group (HSCs in the presence of shRNA-NC or vector); ^#^*p* < 0.05 vs. the pcDNA3-NEAT1 + shRNA-NC or pcDNA3-NEAT1 + miR-NC group (HSCs in the presence of pcDNA3-NEAT1 + shRNA-NC or pcDNA3-NEAT1 + miR-NC). **(K)** Quantitative analysis on α-SMA and Col1A1 immunofluorescence intensity in primary HSCs after transfection; *p < 0.05 vs. the blank (HSCs without treatment) or pcDNA3-NEAT1 + shRNA-NC group (HSCs in the presence of pcDNA3-NEAT1 + shRNA-NC); ^#^*p* < 0.05 vs. the pcDNA3-NEAT1 + miR-NC group (HSCs in the presence of pcDNA3-NEAT1 + miR-NC). All data were measurement data and expressed as mean ± standard deviation. Comparison between two groups was analyzed using unpaired *t*-test. One-way analysis of variance was used for data comparison among multiple groups. The experiment was independently repeated three times. *N* = 6 mice per group.

Next, we investigated the role of NEAT1 in the process of liver fibrosis. RT-qPCR showed that NEAT1 expression was increased in the liver and primary HSCs from NASH mice (*p* < 0.05, Figures 7A–C). In HSCs, silencing NEAT1 could significantly upregulate miR-129-5p expression and downregulate PEG3 expression (*p* < 0.05, Figure 7D). Moreover, NEAT1 silence significantly suppressed cell proliferation and the expression of α-SMA and Col1A1 (*p* < 0.05, Figures 7E–J) in HSCs, while restoring NEAT1 induced cell proliferation and the expression of α-SMA and Col1A1 (*p* < 0.05, Figures 7E–J). In addition, miR-129-5p mimic blocked the enhanced HSC proliferation and higher expression of α-SMA and Col1A1 induced by NEAT1 (*p* < 0.05, Figures 7E–J). Consistently, silencing PEG3 inhibited the elevated HSC proliferation and the expression of α-SMA and Col1A1 induced by NEAT1 (*p* < 0.05, Figures 7E–J). Meanwhile, immunostaining also revealed that miR-129-5p mimic or PEG3 silencing could block the increased expression of α-SMA and Col1A1 induced by NEAT1 (*p* < 0.05, Figure 7K). Therefore, NEAT1 could activate HSCs through regulating the miR-129-5p/PEG3 axis.

### miR-129-5p/PEG3 Axis Regulates HSC Apoptosis Through the NF-κB Signaling Pathway

**FIGURE 8:**
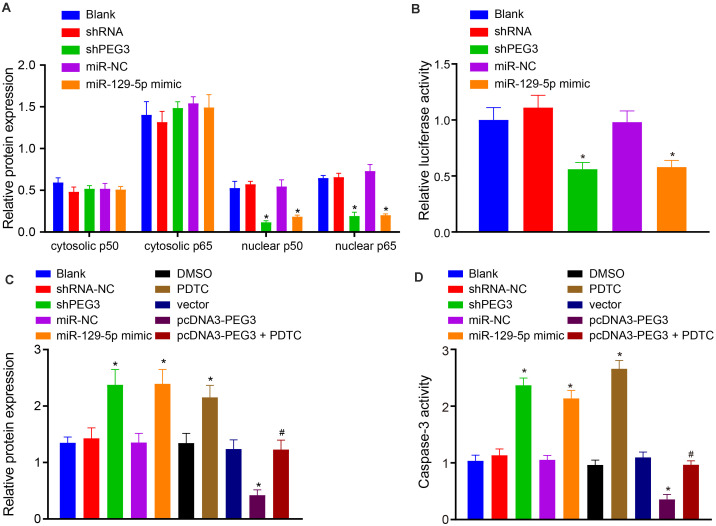
Restoration of miR-129-5p or silencing PEG3 promotes HSC apoptosis through the NF-κB signaling pathway. **(A)** Relative protein levels of p50 and p60 in the nucleus and cytoplasm normalized to GAPDH determined by western blot analysis. **(B)** Relative luciferase activity of NF-κB signaling pathway reporter. **(C)** Relative expression of cleaved PARP normalized to GAPDH determined by western blot analysis. **(D)** Caspase-3 activity. *p < 0.05 vs. the blank, miR-NC, DMSO, or vector group (HSCs without any treatment or in the presence of miR-NC, DMSO, or vector). ^#^*p* < 0.05 vs. the pcDNA3-PEG3 group (HSCs in the presence of pcDNA3-PEG3). All data were measurement data and expressed as mean ± standard deviation. One-way analysis of variance was used for data comparison among multiple groups. The experiment was independently repeated three times. *N* = 6. PARP, poly ADP-ribose polymerase; DMSO, dimethyl sulfoxide.

It is acknowledged that PEG3 regulates cell apoptosis in various cell types through mediating mitochondria translocation of B-cell lymphoma-2 (Bcl-2)-associated protein X (Bax) (Deng and Wu, 2000). On the other hand, anti-apoptotic effects of PEG3 can be achieved through interaction with TRAF2 involving the activated NF-κB signaling pathway (Relaix et al., 1998). Therefore, we intended to explore whether PEG3 could regulate HSC apoptosis through the NF-κB signaling pathway. Results showed that delivery of shPEG3 or miR-129-5p mimic provoked lower expression of p50 or p65 in the HSC nucleus, indicating downregulation of the NF-κB signaling pathway (*p* < 0.05, Figure 8A). Meanwhile, dual-luciferase reporter assay identified the suppressive effects of shPEG3 or miR-129-5p mimic on luciferase activity of the NF-κB pathway reporter (*p* < 0.05, Figure 8B). The activity of the apoptosis enzyme caspase-3 and the level of cleaved poly ADP-ribose polymerase (PARP) could be promoted by shPEG3, miR-129-5p mimic, or PDTC (the NF-κB inhibitor). Moreover, PEG3 overexpression reduced the activity of caspase-3 and the level of cleaved PARP (*p* < 0.05), which could be partially rescued by treatment with a NF-κB inhibitor (*p* < 0.05, Figures 8C,D). Therefore, we infer that the miR-129-5p/PEG3 axis is responsible for HSC apoptosis through the NF-κB signaling pathway.

### Silencing PEG3 Impedes Liver Fibrosis in Mice With NASH Under Positive Mediation of NEAT1

**FIGURE 9:**
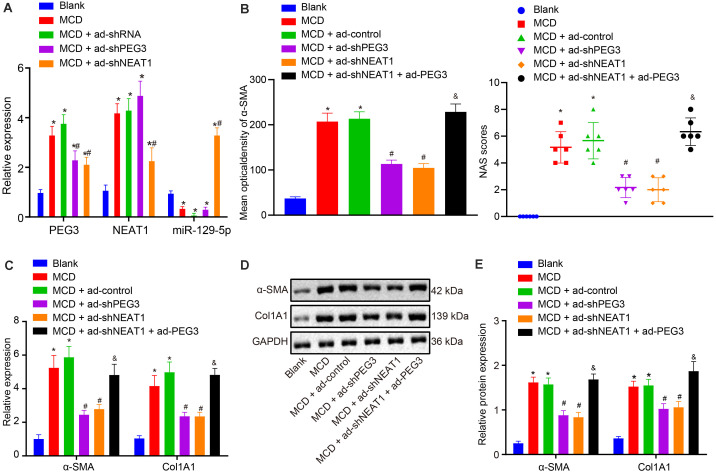
NEAT1 expression has a positive correlation with the effects of PEG3 in NASH mice. **(A)** Relative expression of PEG3, NEAT1, and miR-129-5p determined by RT-qPCR. **(B)** Pathological changes of liver tissue in mice on normal diet and the 5-week MCD diet detected by Masson staining (×200) (the upper diagram) and HE staining (×200) (the lower diagram). **(C)** Relative expression of α-SMA and Col1A1 determined by RT-qPCR. **(D,E)** Relative expression of α-SMA and Col1A1 normalized to GAPDH determined by western blot analysis. *p < 0.05 vs. the blank group (mice on normal diet). ^#^*p* < 0.05 vs. the MCD group (mice on MCD diet alone). ^&^*p* < 0.05 vs. the MCD + ad-shNEAT1 group (mice on MCD diet and injected with ad-shNEAT1). All data were measurement data and expressed as mean ± standard deviation. One-way analysis of variance was used for data comparison among multiple groups. The experiment was independently repeated three times. *N* = 6 mice per group.

For verification of the role of PEG3 in liver fibrosis in mice with NASH, ad-shNEAT1 and/or ad-shPEG3 were injected into NASH mice via their tails and their effects on the progression of liver fibrosis were observed. RT-qPCR showed that treatment with either ad-shNEAT1 or ad-shPEG3 significantly downregulated the expression of NEAT1 or PEG3, respectively, and that ad-shNEAT1 promoted miR-129-5p expression, while inhibiting PEG3 expression (*p* < 0.05, Figure 9A). Masson staining and HE staining showed that the accumulation of collagen in NASH mice could be reversed by silencing PEG3 or NEAT1 (*p* < 0.05, Figure 9B). Circumstantial evidence from RT-qPCR and western blot analysis suggested that silencing PEG3 or NEAT1 inhibited the expression of α-SMA and Col1A1 in liver tissues from NASH mice. Additionally, compared with ad-shNEAT1 treatment alone, combined injection of ad-shNEAT1 and ad-PEG3 generated much more hepatic collagen and higher expression of α-SMA and Col1A1 in mice on MCD diet (*p* < 0.05, Figures 9C–E). Taken together, we conclude that liver fibrosis of mice can be suppressed by silencing NEAT1 through inhibiting PEG3 expression.

## Discussion

Non-alcoholic steatohepatitis is an important form of NAFLD, which can arise as an inflammatory response leading to hepatocellular injury (Verna, 2017). Biopsy is the most widely used method for diagnosis and staging of NASH, but it is invasive and only applicable in limited populations, thus calling for novel diagnostic methods (Vernon et al., 2011). Intriguingly, lncRNAs have attracted attention in this regard because of their significant functions in liver diseases, including NAFLD (Chen, D. L. et al., 2017). The current study concluded that silencing NEAT1 expression could enhance the negative regulation of PEG3 by miR-129-5p and effectively reduce the fibrotic characteristics of HSCs in the setting of NASH involving the NF-κB signaling pathway.

A key finding of the present study consists of the observation that NEAT1 and PEG3 were highly expressed, whereas miR-129-5p had poor expression in liver tissue and HSCs from NASH mice. Consistent with this, Leti et al. (2017) have observed upregulated NEAT1 in fibrosis of NASH when compared with normal counterparts. Besides, lncRNAs possess the ability to form specific binding with miRNAs, and miRNA motifs mediate the expression levels of protein-coding genes (Zhang et al., 2016). We now report competitive binding between NEAT1 and miR-129-5p, such that miR-129-5p can negatively regulate its target gene, PEG3. Accumulating evidence shows lncRNAs can competitively bind to miRNAs in the cytoplasm, further impairing the miRNA-mediated inhibition of their target genes (Chen, Y. et al., 2017; Wang et al., 2017). A similar regulatory mechanism has been reported that lncRNA prostate cancer-associated transcript 1 interacts with miR-129-5p in HCC and exerts derepression effects on miR-129-5p (Zhang et al., 2018). An interaction between NEAT1 and miR-129-5p has been reported in different types of cancer. For instance, NEAT1 could inhibit papillary thyroid cancer progression by upregulating miR-129-5p (Zhang et al., 2018). NEAT1 promotes the proliferation of HCC cells in a manner dependent on miR-129-5p (Fang et al., 2017). Also, NEAT1 expression was negatively correlated with miR-129-5p expression in HCC (Shaker et al., 2019). All these findings support our result that NEAT1 could competitively bind to miR-129-5p.

Depletion of lncRNAs can downregulate their neighboring protein-coding genes in association with the occurrence and progression of certain diseases (Sun et al., 2015). Importantly, in our study, silencing PEG3 or NEAT1 or restoring miR-129-5p tended to impede liver fibrosis *in vivo* and suppress activation of HSCs *in vitro*. These effects were evidenced by higher expression levels of caspase-3 and cleaved PARP as well as lower expression levels of α-SMA and Col1A1, which involved the inactivation of the NF-κB signaling pathway. Furthermore, these alterations could be reversed by upregulation of NEAT1.

The activation of HSCs, also known as trans-differentiation, is a leading attributor of liver fibrogenesis (Higashi et al., 2017). In the process of liver fibrogenesis, HSCs can activate or transdifferentiate into myofibroblasts-like cells characterized by contractility and fibrogenesis (Jang et al., 2015). α-SMA is defined as an actin isoform, the expression of which is localized in fibro-contractive lesions and healing wounds, such that elevated α-SMA expression is also indicative of HSC activation (Barbero-Becerra et al., 2015). Similarly, the miR-21 inhibitor diminished the expression of α-SMA and Col1A1 in activated HSCs, by a mechanism with demonstrated therapeutic potential in liver fibrosis (Fu et al., 2017). Moreover, in human fibrotic liver samples, liver fibrosis and HSC activation show a positive correlation with NEAT1 expression (Yu et al., 2015). Caspase-3 splits PARP to induce DNA fragment and apoptosis, and activation of caspase-3 can function as a marker for HSC apoptosis (Chen et al., 2016). Additionally, blocking NF-κB activation is functionally characterized to suppress HSC activation for potential alleviation of liver fibrosis (Zhang et al., 2016). As a critical redox-sensitive transcription factor contributing to inflammatory response, inhibition of NF-κB can achieve protective effects in NAFLD hepatocytes (Xiao et al., 2014).

**FIGURE 10:**
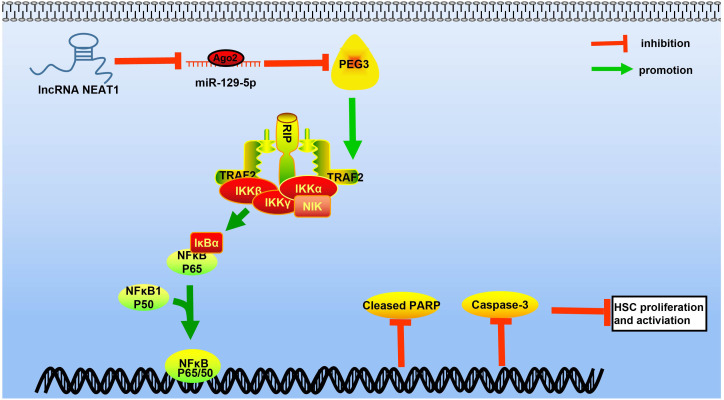
Diagram depicting the molecular regulation mechanism of the NEAT1/miR-129-5p/PEG3 axis in liver fibrosis of mice with NASH through the NF-κB signaling pathway. In mice with NASH, NEAT1, and PEG3 are highly expressed while miR-129-5p is poorly expressed. NEAT1 upregulates PEG3 expression by competitively binding to miR-129-5p to activate the NF-κB signaling pathway, by which HSC apoptosis is inhibited and HSCs are activated to accelerate the process of liver fibrosis. *N* = 6.

Present findings give significant new evidence that NEAT1 binds to miR-129-5p and that competitive inhibition of NEAT1 or PEG3 expression by miR-129-5p can alleviate liver fibrosis via effects on the NF-κB signaling pathway (Figure 10).

## Conclusion

Our study has highlighted the value of NEAT1 as a potential prognostic biomarker and/or therapeutic target in NASH. However, future research should investigate off-target effects of mutant of NEAT1 or PEG3 on miR-129-5p expression, as a precondition for eventual clinical translation. Also, previous studies made use of two animal models to ensure generalizability, such that the present use of a single MCD model may be a limitation of this study. Besides, this study only addresses the roles of NEAT1/miR-129-5p/PEG3 axis in NASH in animal experiments, and the present lack of corresponding knowledge in humans must be addressed in future research.

## Data Availability Statement

The original contributions presented in the study are included in the article/Supplementary Material, further inquiries can be directed to the corresponding author/s.

## Ethics Statement

The animal study was reviewed and approved by the Institutional Animal Care and Use Committee of the Fifth Affiliated Hospital, Southern Medical University.

## Author Contributions

ZZ designed the study. HW collated the data. BP carried out data analyses. JW produced the initial draft of the manuscript. FZ contributed to drafting the manuscript. All authors have read and approved the final submitted manuscript.

## Conflict of Interest

The authors declare that the research was conducted in the absence of any commercial or financial relationships that could be construed as a potential conflict of interest.

## Footnotes

1 http://biogps.org/#goto=welcome

2 http://www.ncbi.nlm.nih.gov/geo

3 http://34.236.212.39/microrna/home.do

4 http://129.206.7.150/

5 http://www.targetscan.org/vert_71/

## Supplementary Material

Supplementary Figure 1 Relative expression of NEAT1 and PEG3 in the mimic-NC and miR-129-5p mimic groups determined by RT-qPCR.
