# Supplementary figures and images for: Downregulated microRNA-129-5p by Long Non-coding RNA NEAT1 Upregulates PEG3 Expression to Aggravate Non-alcoholic Steatohepatitis
Source: Front Genet. 2021 Jan 26;11:563265. doi: 10.3389/fgene.2020.563265 (PMC7870803; doi:10.3389/fgene.2020.563265)

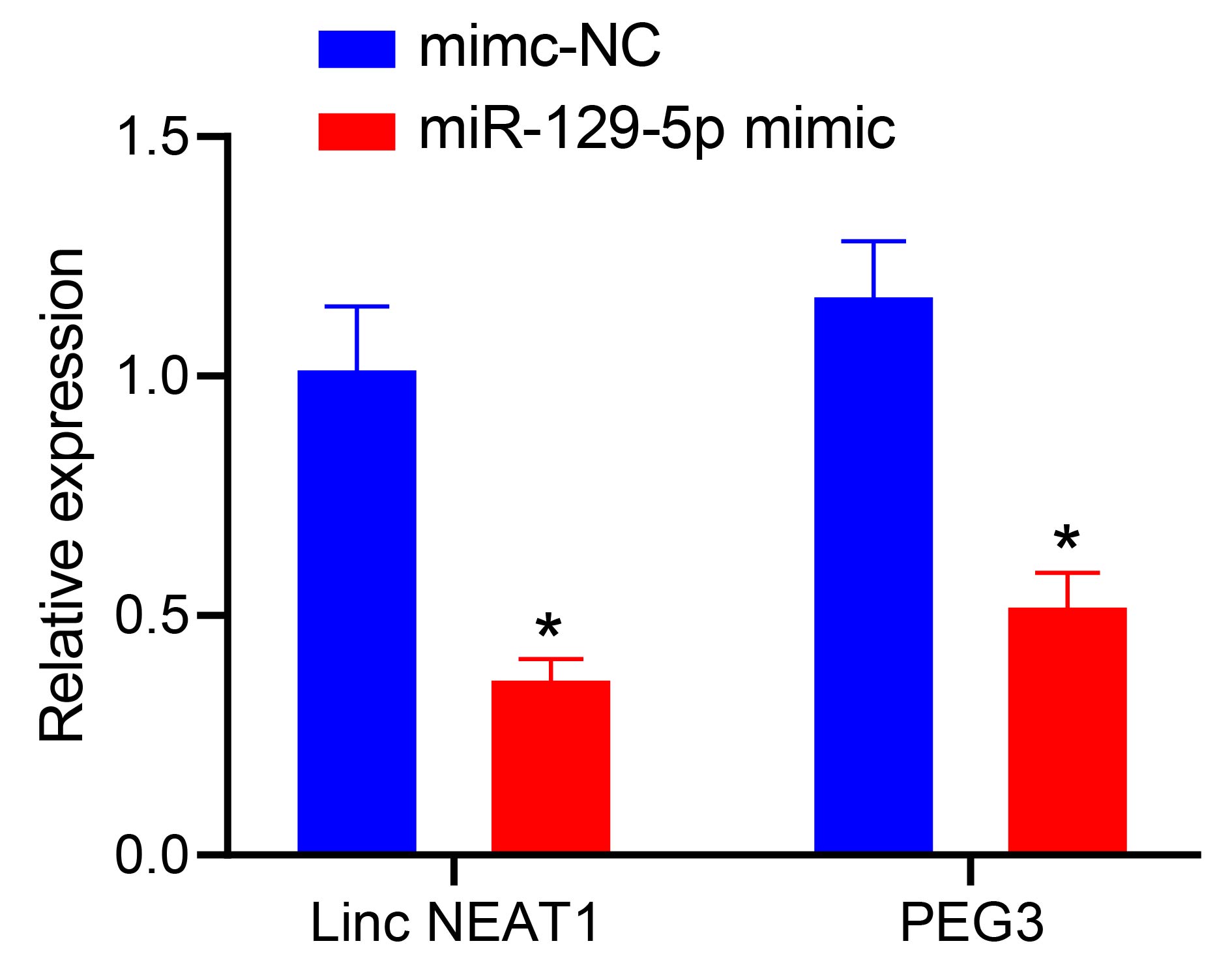

Supplement: Supplementary Figure 1 — Relative expression of NEAT1 and PEG3 in the mimic-NC and miR-129-5p mimic groups determined by RT-qPCR. [file Image_1.JPEG]
